# The Coadministration of Levosimendan and Exenatide Offers a Significant Cardioprotective Effect to Isolated Rat Hearts against Ischemia/Reperfusion Injury

**DOI:** 10.3390/jcdd9080263

**Published:** 2022-08-12

**Authors:** Vasileios Leivaditis, Efstratios Koletsis, Nikolaos Tsopanoglou, Nikolaos Charokopos, Cristian D’Alessandro, Konstantinos Grapatsas, Efstratios Apostolakis, Effrosyni Choleva, Maria Plota, Andreas Emmanuil, Manfred Dahm, Dimitrios Dougenis

**Affiliations:** 1 Department of Cardiothoracic and Vascular Surgery, Westpfalz-Klinikum, Hellmut-Hartert-Strasse 1, 67655 Kaiserslautern, Germany; 2 Department of Cardiothoracic Surgery, University Hospital of Patras, 26504 Patras, Greece; 3 Department of Pharmacology, School of Medicine, University of Patras, 26504 Patras, Greece; 4 Laboratory of Biomechanics & Biomedical Engineering, Department of Mechanical Engineering & Aeronautics, University of Patras, 26504 Patras, Greece; 5 Department of Thoracic Surgery, Medical Center-University of Freiburg, Faculty of Medicine, 79106 Freiburg, Germany; 6 Department of Cardiothoracic Surgery, University Hospital of Ioannina, 45500 Ioannina, Greece; 7 Laboratory of Molecular Pharmacology, Department of Pharmacy, University of Patras, 26504 Patras, Greece; 8 Department of Microbiology, School of Medicine, University of Patras, 26504 Patras, Greece; 9 Laboratory of Hematology, University Hospital of Patras, 26504 Patras, Greece; 10 Department of Cardiothoracic Surgery, Attikon University Hospital of Athens, 12462 Athens, Greece

**Keywords:** Levosimendan, Exenatide, ischemia-reperfusion injury, isolated rat heart, cardioprotection

## Abstract

(1) Background: The present study aims to investigate the effect of administration of Levosimendan and Exenatide in various concentrations, as well as of the coadministration of those agents in an ischemia–reperfusion injury isolated heart model. (2) Methods: After 30 min of perfusion, the hearts underwent a 30 min period of regional ischemia followed by a 120 min period of reperfusion. All animals were randomly divided into 12 experimental groups of nine animals in each group: (1) Control, (2) Sham, (3) Digox (Negative control, Digoxin 1.67 μg/min), (4) Levo 1 (Levosimendan 0.01 μg/min), (5) Levo 2 (Levosimendan 0.03 μg/mL), (6) Levo 3 (Levosimendan 0.1 μg/min), (7) Levo 4 (Levosimendan 0.3 μg/min), (8) Levo 5 (Levosimendan 1 μg/min), (9) Exen 1 (Exenatide 0.001 μg/min), (10) Exen 2 (Exenatide 0.01 μg/min), (11) Exen 3 (Exenatide 0.1 μg/min) and (12) Combi (Levosimendan 0.1 µg/mL + Exenatide 0.001 μg/min). The hemodynamic parameters were recorded throughout the experiment. Arrhythmias and coronary flow were also evaluated. After every experiment the heart was suitably prepared and infarct size was measured. Markers of myocardial injury were also measured. Finally, oxidative stress was evaluated measuring reactive oxygen species. (3) Results: A dose-dependent improvement of the haemodynamic response was observed after the administration of both Levosimendan and Exenatide. The coadministration of both agents presented an even greater effect, improving the haemodynamic parameters further than the two agents separately. Levosimendan offered an increase of the coronary flow and both agents offered a reduction of arrhythmias. A dose-dependent reduction of the size of myocardial infarction and myocardial injury was observed after administration of Levosimendan and Exenatide. The coadministration of both agents offered a further improving the above parameters. Levosimendan also offered a significant reduction of oxidative stress. (4) Conclusions: The administration of Levosimendan and Exenatide offers a significant benefit by improving the haemodynamic response, increasing the coronary flow and reducing the occurrence of arrhythmias, the size of myocardial injury and myocardial oxidative stress in isolated rat hearts.

## 1. Introduction

Despite the vast improvement of the treatment of myocardial infarction and its consequences, ischemia-reperfusion injury remains a very common challenge in the clinical practice of all cardiovascular specialties and further therapeutic strategies are being developed aiming to its further reduction.

Levosimendan is nowadays a well-known calcium sensitizer. It increases the sensitivity of the myocardial cells to calcium and manages to increase cardiac contractility without causing a rise in intracellular calcium. Three key mechanisms of action which are responsible for its positive inotropic, vasodilatatory and cardioprotective effects [1]: i. Calcium sensitization by selective binding to calcium-saturated cardiac troponin C increases the contractile force of the cardiac myocytes without affecting relaxation [2,3]. ii. Opening of potassium-ATP (K_ATP_) channels in vascular smooth muscle cells elicits both arterial and venous vasodilation as well as improvement in coronary artery circulation [4,5]. iii. Opening of K_ATP_ channels in the mitochondria of cardiomyocytes achieves a cardioprotective effect in situations when the heart is subjected to ischemic events [6,7]. Moreover, the fact that Levosimendan can prevent or limit myocyte apoptosis via the activation of mitochondrial K_ATP_ channels provides a potential mechanism whereby this agent might protect cardiac myocytes during episodes of acute heart failure as well as in chronic heart failure [8,9]. Several studies over the last decade have demonstrated the cardioprotective effect of Levosimendan [10,11,12,13,14,15].

Exenatide is also a well-known treatment used for type 2 diabetes mellitus as an add-on to the classic antidiabetic therapies. Exenatide binds to the intact human Glucagon-like peptide-1 receptor (GLP-1R) in a similar way to the human peptide glucagon-like peptide-1 (GLP-1) [16]. Several studies have shown that GLP-1 based therapies could play an important role in the regulation of cardiovascular function after ischemia and reperfusion. This was partly mediated by the stimulation of myocardial glucose uptake; however, the exact underlying mechanism still remains unclear [17,18,19]. Targeting the balance between fatty acid b-oxidation and glucose oxidation could optimize cardiac energy substrate metabolism. This could be an effective therapeutic intervention to maintain contractile activity in acute cardiac pathological conditions [19,20].

In this study, we investigated the dependence between the administrated doses of Levosimendan and Exenatide separately and the outcome of the hemodynamic response of the heart in an isolated rat heart model of ischemia-reperfusion injury. After determining the optimal number of doses of both agents providing the maximal effect, we combined the administration of these agents and compared the effect of the combination to the effect of the administration of every agent separately.

## 2. Materials and Methods

*Animal Cohort*. The study was approved by the Institutional Committee of Animal Care of the University of Patras, Greece and the Prefecture of Western Greece (Protocol Nr. 261936/1076). The total animal cohort comprised 108 male Wistar rats (20–24 weeks of age, 350–400 g weight). All animals received humane care, according to European legislation (European Union directive for the protection of animals used for scientific purposes, 2010/63/EU and European Animal Research Association) and the Consensus Author Guidelines on Animal Ethics and Welfare for Veterinary Journals; they were housed 1–2 per cage, under optimal laboratory conditions (controlled temperature, humidity, and 12: 12 h-light: dark cycles), with free access to water and standard rodent chow. Appropriate statistical analysis software (powerandsamplesize.com (accessed on 3 April 2017)) was used to estimate the required number of experimental animals. After setting a statistical power of 80% and a type A error of 5% and estimating the rate of improvement of the haemodynamic parameters in the control group and in the factor administration groups, it was estimated that 9 experimental animals per group would be needed. Therefore, the total number of rats needed to complete the proposed protocol was estimated to be 108 rats.

**Figure 1:**
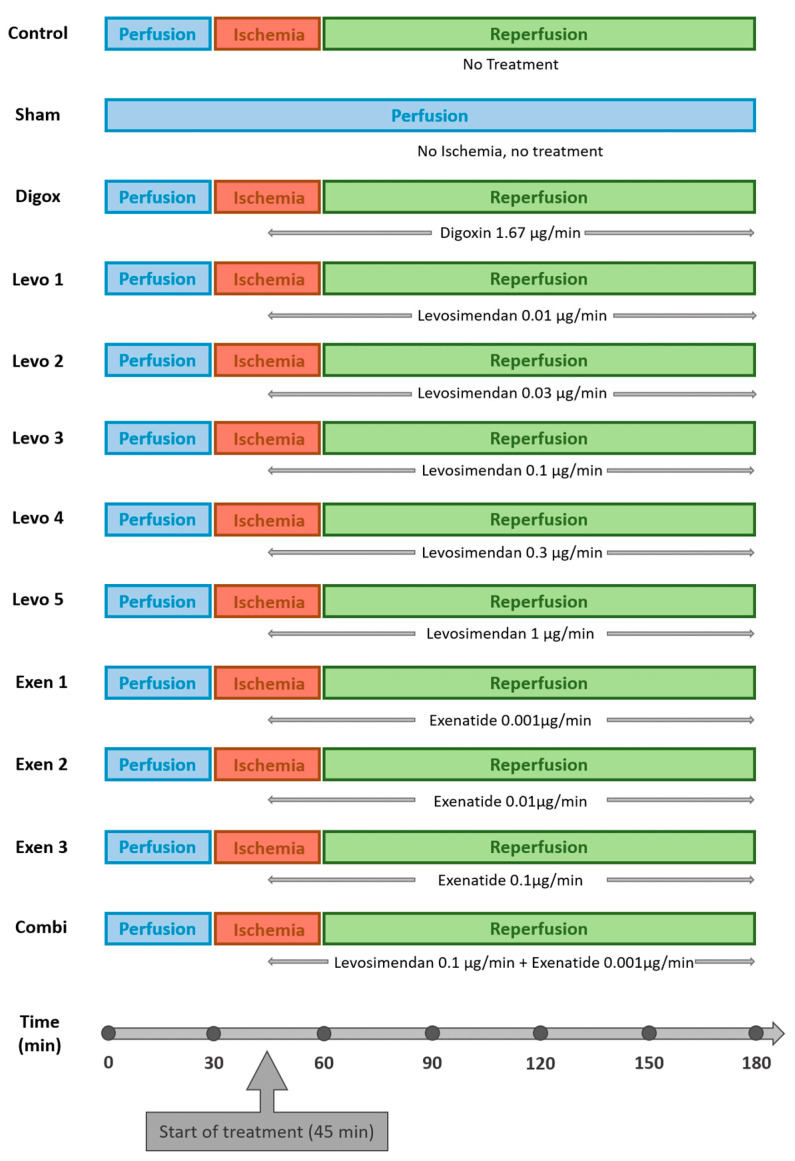
Schematic demonstration of the experimental protocol showing the groups and the durations of the three phases of the experiment (perfusion, ischemia and reperfusion). The application of the treatment started at the middle of the ischemia phase and was continued until the end of the reperfusion phase.

*Heart preparation*. The Langendorff-perfused isolated heart method offers a very reliable model for the analysis of contractile function and responses to ischemic insult [21,22,23,24]. All rats were anesthetized with pentobarbital sodium (50 mg/kg ip), Ketamine (75 mg/kg ip) and Xylazine (10 mg/kg ip). The inferior caval vein was exposed through median laparotomy and the rats were systematically treated with heparin sodium (1000 IU/kg) to avoid clot formation. After median sternotomy, hearts were fast excised from the rat chest and put into ice-cold Krebs-Henseleit (KH) solution to stop contractions. Hearts were then hung to the isolated perfusion apparatus and were retrogradely perfused via a cannula in the ascending aorta in a nonworking “Langendorff” preparation model at a constant pressure of 75 mmHg. The perfusion fluid had a stable pH of 7.4. KH solution and was used as a perfusate, containing 120 mM NaCl, 25 mM NaHCO_3_, 5.9 mM KCl, 1.2 mM MgSO_4_, 2.25 mM CaCl_2_, 1.2 mM KH_2_PO_4_ and 11 mM glucose. The perfusate was bubbled with Carbozen gas mixture (95% O_2_ and 5% CO_2_). The temperature of the perfusate solution and the heart chamber was maintained stable at 38 °C with a thermostatically controlled water-circulating system. [21,22,23,24]. A water-filled balloon fixed to a pressure transducer was inserted through the left atrium and the mitral valve into the left ventricle (LV) for determination of LV systolic pressure (LVSP) and end-diastolic pressure (LVEDP). LVEDP was adjusted to 5 mmHg before the start of the experiment by adjustment of the volume in the intraventricular balloon. In contrast to other methods of recording the hemodynamic response, such as the use of a force transducer attached to the apex or an intraventricular catheter, the isovolumetric condition obtained by the intraventricular balloon standardizes the situation with respect to preload and afterload [25]. The heart was initially perfused and stabilized for 30 min. After that, a regional ischemia of the anterior wall was induced through a ligation of the left anterior descending coronary artery (LAD). The LAD was encircled with a 6–0 Polypropylene suture near its origin, extending from the pulmonary cone to the myocardium under the left atrial appendage. The application of the suture following these specific anatomical landmarks ensures comparable myocardial ischemic area in all hearts. The suture was then passed through a tourniquet. Tightening and release of the tourniquet induced ischemia and reperfusion respectively. After an ischemia of 30 min, the coronary artery was opened and the heart was reperfused for 120 min. The hearts were randomly assigned to a control group (*n* = 9) where no treatment was applied, a sham group (*n* = 9) where no ischemia was applied, an negative control group (Digox, *n* = 9) with administration of Digoxin (Anfarm Hellas S.A., Kifisia 14564, Greece) with a perfusion rate of 1.67 μg/min and one of the nine experimental groups (*n* = 9 per group): administration of Levosimendan (Orion Pharma Hellas Sole Shareholder Co. Ltd., Alimos 17455, Greece) with a perfusion rate of 0.01 μg/min (Levo 1), Levosimendan with a perfusion rate of 0.03 μg/min (Levo 2), Levosimendan with a perfusion rate of 0.1 μg/min (Levo 3), Levosimendan with a perfusion rate of 0.3 μg/min (Levo 4), Levosimendan with a perfusion rate of 1 μg/min (Levo 5), Exenatide (AstraZeneca S.A., Athens 15123, Greece) with a perfusion rate of 0.001 μg/min (Exen 1), Exenatide with a perfusion rate of 0.01 μg/min (Exen 2), Exenatide with a perfusion rate of 0.1 μg/min (Exen 3) and coadministration of Levosimendan and Exenatide with a perfusion rate of 0.1 µg/mL and 0.001 μg/min, respectively (Combi). The administration of all additional pharmacologic agents started at 15 min of ischemia (in the middle of the ischemia phase) and was continued until the end of the experiment. The administration of the treatment lasted 135 min until the end of the reperfusion phase. The groups and the experimental protocol are presented in Figure 1.

*Assessment of LV function*. The haemodynamic parameters such as left ventricular systolic pressure (LVSP), left ventricular end-diastolic pressure (LVEDP), left ventricular developed pressure (LVDP, defined as peak systolic pressure minus end-diastolic pressure, LVDP = LVSP − LVEDP), heart rate (HR), rate pressure product (RPP = HR × VSP), maximum positive (+dP/dt) and negative (−dP/dt) values of the first derivative of pressure were recorded throughout the experiment [21,22,23,24,25,26]. Ischemia-induced arrhythmias and coronary blood flow (CBF) were also measured in all animal hearts. The data acquisition was done with a Labjack U3HV data logger (Labjack, LabJack Corporation 6900 West Jefferson Ave Suite 110 Lakewood, CO 80235, USA) coupled to a pressure transducer amplifier (NL108A, Digitimer, 37 Hydeway Welwyn Garden City Hertfordshire AL7 3BE UK). The custom-made pressure balloon was connected directly to the disposable pressure transducer, which was coupled with the pressure amplifier. The output of the amplifier was later acquired and digitalized with the data logger at a rate of 500 samples per second. The system was primed and calibrated using a water column and measuring the output signal of the sensor in the range between 10 to 50 cmH_2_O. Once calibrated, the balloon was connected to the sensor and introduced into the left ventricle. The data was then acquired until the end of the experiment. The gathered data were analyzed using Matlab (Matlab, The MathWorks, Inc., 1 Apple Hill Drive Natick, MA 01760-2098 USA). The raw data were pre-processed to obtain the above mentioned haemodynamic parameters. All these parameters were registered for 12 timepoints of the experiment (15, 30, 45, 60, 75, 90, 105, 120, 135, 150, 165 and 180 min, respectively).

*Measurement of coronary flow*. Effluent from the venous return can easily be collected since fluid from the coronary sinus will rise to the surface of the preparation through the openings of the vena cava or pulmonary artery. The heart chamber of the left ventricle is empty or sparsely filled with fluid from the thebesian veins [21,22,24]. In order to estimate the coronary flow rate, the effluent of the perfusate was collected for 2 min at 12 different timepoints of the experiment (namely, 15, 30, 45, 60, 75, 90, 105, 120, 135, 150, 165 and 180 min respectively), and the volume was measured. Coronary flow was calculated and reported as milliliters per minute (mL/min).

*Estimation of arrhythmia scores*. During the 30 min of ischemia and the 120 min of reperfusion, ventricular arrhythmias were recorded and evaluated according to the Lambeth convention [27]. All arrhythmias were recorded and categorized as ventricular ectopic beats (VEB) ventricular tachycardias (VT) and ventricular fibrillations (VF). In order to estimate the severity of the recorded arrhythmias, the onset time and the duration of arrhythmias were evaluated. The grade of the severity was estimated using the following scoring system [28]. 0: <10 ventricular premature beats, 1: ≥10 ventricular premature beats, 2: VT (duration < 30 s), 3: VT (duration ≥ 30 s), 4: VF starting 15 min after the onset of ischemia, 5: VF starting 5–15 min after the onset of ischemia and 6: VF starting within 5 min after the onset of ischemia. In our study, the score was partially modified and we started recording arrhythmias at the point of the initiation of the administration of the pharmacological agent after 15 min of ischemia, namely 5, 5–15 and >15 min after starting the application of the treatment so that we could achieve a better correlation to the effect of the treatment.

*Measurement of Troponin I*. The ARCHITECT STAT High Sensitive cardiac Troponin-I (cTnI) assay was used [29,30,31]. It is a two-step immunoassay to determine the presence of cTnI in plasma and serum using chemiluminescent microparticle immunoassay (CMIA) technology with flexible assay protocols, referred to as Chemiflex.

*Measurement of Myocardial Creatine kinase (CK-MB)*. Creatine kinase catalyzes the reaction between creatine phosphate and ADP with formation of creatine and ATP. The ATP, formed in the presence of glucose and hexokinase (HK), yields ADP and glucose-6-phosphate. The glucose-6-phosphate formed in the presence of glucose-6-phosphate dehydrogenase (G6P-DH) reacts with β-NADP+ forming 6-phosphogluconate and β-NADPH. The presence of mouse antibodies that inhibit CK-MM activity in the reaction mixture allows the determination of the residual activity of CK-B isoenzymes (CK-MB and CK-BB). The CK-MB activity is obtained by multiplying the CK-B activity by two. By measuring the variation of the absorbance due to transformation of β-NADP+ into β-NADPH in a time interval at 340 nm, it is possible to calculate the residual activity in the examined sample [29,32]. Methodology: IFCC Method/Immunoinhibition. Analyzer: ABBOTT/Architect c8000.

*Measurement of Lactate dehydrogenase (LDH)*. Lactate dehydrogenase is a hydrogen transfer enzyme that catalyzes the oxidation of L-lactate to pyruvate with the mediation of NAD+ as a hydrogen acceptor. This method uses the IFCC recommended 2–4 forward reaction—Lactate to Pyruvate [29,33].

The above markers of myocardial injury (TnI, CK-MB and LDH) were measured in the effluent of the hearts. The samples were taken at the end of the ischemia phase and an hour after the beginning of the reperfusion (in the middle of the reperfusion phase).

**Figure 2:**
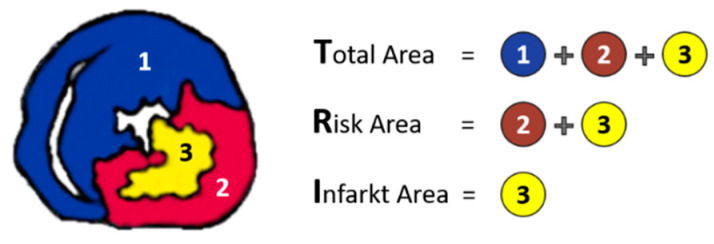
Schematic representation of the heart section with the measured areas. T = total area of the section, R = area at risk (area perfused by the LAD) and I = infarct area.

*Estimation of the size of myocardial infarction*. Infarct size was expressed as a percentage of ischemic myocardial area to the area at risk. In order to obtain a precise measurement of the infarct area and the area at risk, we used a common coloring method [34,35,36,37,38]. A total of 5 mL of 1% 2, 3, 5-triphenyltetrazolium chloride (TTC) phosphate buffer (37 °C, pH = 7.4) was administered to the hearts via the aortic cannula. Then, the heart was incubated in the 1% TTC solution for 10 min. After that, the LAD was closed by the tourniquet. Then, 3 mL of 2.5% Evans Blue solution, diluted in distilled water, was infused through the aortic cannula. Finally, the heart was washed and frozen at −20 °C for 24 h. Then, the hearts were transversely sliced in 2 mm sections from the apex to the base, slightly above the site of ligature of the LAD and parallel to the atrioventricular groove. Six sections were made in every heart. Then, the slices were incubated in 5% formaldehyde solution for 5 h. After placement between glass plates, stereoscopic images of the sections were taken and the risk zone, the infarcted area and the normal myocardium were identified. Sections of the ventricle have uniformly blue areas. After administration and incubation in TTC, viable myocardium stains brick red and the infarct appears pale white. The total area of the heart section (T), the area at risk (R) and the area of infarction (I) were determined by computerised planimetry using an image analysis software program (Image J V 1.8.0, National Institutes of Health and the Laboratory for Optical and Computational Instrumentation LOCI, University of Wisconsin, Madison, WI, USA) as shown in Figure 2.

*Evaluation of oxidative stress*. Homogenization of heart tissue was performed mechanically in a mortar placed on ice. The lysis of the cells was done with the appropriate buffer which contained inhibitors that prevent proteolysis, dephosphorylation and denaturation of the samples, and the whole process was carried out strictly on ice to avoid the destruction of the protein extract. A horizontal incision was made in order to have a representative sample from the entire length of the tissue. The tissue section was placed in an Eppendorf tube and weighed to achieve a final tissue weight of 0.03 g, according to the homogenization protocols. Then, the tissue was transferred to a special homogenizing mortar, where 500 mL of immunoprecipitation lysis buffer (RIPA) solution were added. In order to maximize the amount of protein extract, the tissue was incubated with RIPA solution for 30 min and then crushed. All procedures were performed strictly on ice. RIPA solution contains sodium dodecyl sulfate (SDS) detergent for membrane rupture and protease inhibitors to inhibit protein degradation. The homogenized tissue was transferred to a centrifuge tube. Centrifuging at 20,000× *g* for 30 min at 4 °C followed. The supernatant was transferred to a new sterile Eppendorf tube for the determination of total proteins in the sample by the Bradford method. Accumulation of active oxygen radicals (ROS) was measured with the reagent 5-(and-6)-carboxy-2′,7′-(dichloro-dihydro)-diaketo-fluorescein (carboxy-H2DCFDA, Fluka Chemical, Milwaukee, WI, USA). This is an ester that penetrates the membrane, is non-fluorescent and sensitive to redox. Once this reagent is oxidized by intracellular oxygen radicals, the resulting product, carboxy-DCF, emits green fluorescence. DCF fluorescence was measured in a special instrument called a fluorescence meter [39]. The accumulation of ROS in the extracts that emerged after the homogenization of the heart tissues, was measured here with the DCFDA cellular ROS assay kit according to the following procedure:

1. An amount of the extract corresponding to 50 μg of protein (as determined by the Bradford method) was added to a special 96-microwell plate.
2. The specific carboxy-H2 DCFDA reagent was added to the same microwell at a concentration of Ct = 50 μM in PBS. The reagent was stored in the freezer at Char = 0.015 M covered with foil due to photosensitivity.
3. Incubation for 20 min at room temperature, in the absence of light.
4. Finally, a measurement was made on the fluorometer and the values were recorded per microcell. The fluorescence intensity was determined spectrophotometrically, using as excitation wavelength of 485 nm and emission wavelength of 500 nm.

*Statistical analysis*. Quantitative variables were presented as mean values (SD) and minimum, maximum values. A longitudinal analysis model was used to investigate the effect of time and group. For the comparisons of proportions, chi-square and Fisher’s exact tests were used. Furthermore, in order to examine the differences in mean values of continuous variables, the parametric one-way ANOVA test for independent samples was used to assess for differences. Logistic regression analyses were applied to explore the effect of time and group. Overall, all statistical analyses were performed using STATA v.14 (StataCorp LLC, 4905 Lakeway Drive College Station, Texas 77845-4512, USA). The aforementioned statistical tests were performed at a 0.05 significance level.

## 3. Results

### 3.1. Left Ventricular Systolic Pressure (LVSP)

**Figure 3:**
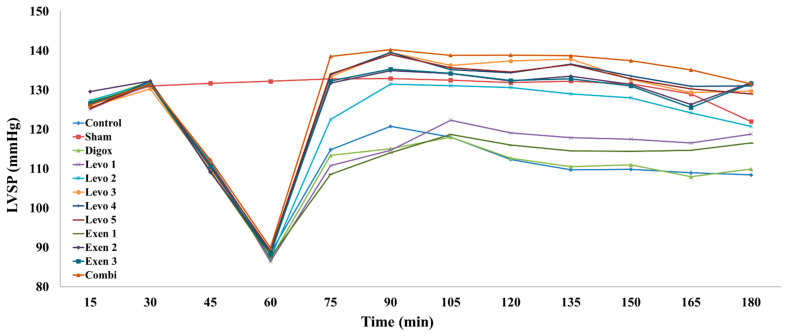
Changes in LVSP across groups over time.

A regression analysis was performed to determine whether there is an association between the LVSP and different types of groups over time. Based on the results, the main effect of time was found to be statistically significant (b = 0.596, *p* < 0.001), specifically, for every additional quarter it was observed 0.596 mmHg increase in LVSP levels. Regarding the groups, it was observed that groups Sham, Levo 2, Levo 3, Levo 4, Levo 5, Exen 2, Exen 3 and Combi present higher LVSP levels compared to the control group (Table 1, Figure 3). In order to examine whether there are any statistically significant differences between the Levo groups and between the Exen groups, extra pairwise comparison was conducted. The results showed that the Levo 1 group presents lower LVSP levels compared to Levo 2 (*p* = 0.003) and Levo 2 presents lower LVSP levels compared to Levo 3 (*p* = 0.007). Levo 4 and Levo 5 did not present any significant improvement of LVDP compared to Levo 3. Exen 1 presents lower LVSP levels compared to Exen 2 (*p* < 0.001). Exen 3 did not present any significant increase of LVSP compared to Exen2. This comparison demonstrates that the maximal effect was achieved in the group Levo 3 for Levosimendan and Exen 2 for Exenatide, respectively. These were therefore considered as the main reference groups for every agent. A further increase of the doses administered in those groups did not present any significant increase of the effect of those agents. A separate regression analysis was performed to determine whether there is association between Combi and Levo 3 and Exen 2 groups over time. Based on the results, the main effect of time was found to be statistically significant (b = 1.530, *p* < 0.001) and, specifically, for every additional quarter, a 1.530 mmHg increase in LVSP levels was observed. Regarding the groups, it was observed that Exen 2 (*p* = 0.050) had lower LVSP levels compared to the Combi group. Combi also presented higher levels compared to Levo 3, but this difference was not significant (*p* = 0.194).

### 3.2. Left Ventricular End-Diastolic Pressure (LVEDP)

**Figure 4:**
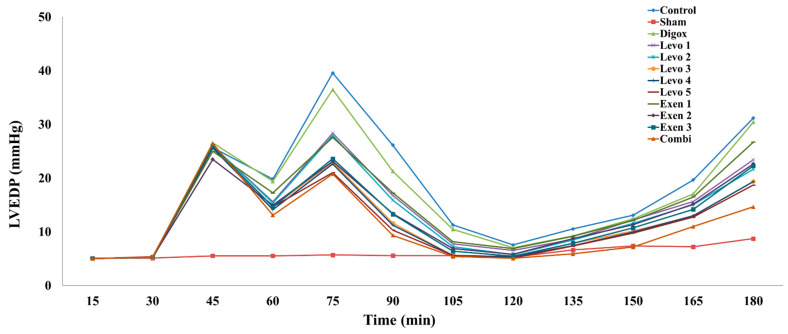
Changes in LVEDP across groups over time.

LVEDP is an important haemodynamic parameter which reflects the stiffness of the left ventricle. The main effect of time was found to be statistically significant (b = 0.248, *p* < 0.001). For every additional quarter, a 0.248 mmHg increase in LVEDP levels was observed. Regarding the groups, it was observed that groups Sham, Levo 1, Levo 2, Levo 3, Levo 4, Levo 5, Exen 1, Exen 2, Exen 3 and Combi had lower LVEDP levels compared to the control group (Table 2, Figure 4) and therefore less myocardial stiffness. The pairwise comparison between the Levo and the Exen groups showed that there were no statistically significant differences between the groups (*p* < 0.999). The regression analysis between the different types of groups over time showed that the main effect of time was found to be non-statistically significant (b = 0.078, *p* = 0.484). Additionally, the Exen 2 group (*p* = 0.019) had higher LVEDP levels compared to the Combi group. Levo 3 also presented higher levels of LVEDP, but these were, however, statistically insignificant (*p* = 0.128).

### 3.3. Left Ventricular Developed Pressure (LVDP)

**Figure 5:**
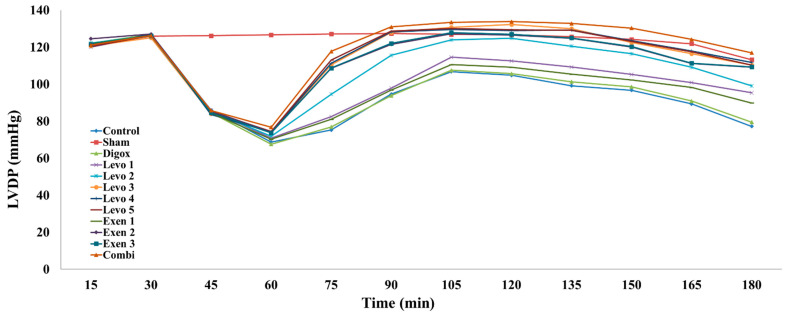
Changes in LVDP across groups over time.

The LVDP is the most significant haemodynamic parameter in isolated heart models as it does more accurately demonstrate the haemodynamic performance of the heart. Concerning the LVDP values, the main effect of time was found to be statistically significant (b = 0.348, *p* = 0.011); specifically, for every additional quarter, a 0.348 mmHg increase in LVDP levels was observed. Sham, Levo 1, Levo 2, Levo 3, Levo 4, Levo 5, Exen 2, Exen 3 and Combi had higher LVDP levels compared to the control group (Table 3, Figure 5). The pairwise comparison showed that Levo 1 presented lower LVDP levels compared to Levo 2 (*p* = 0.001), Levo 2 presented lower LVDP levels compared to Levo 3 (*p* = 0.008) and the Exen 1 group presented lower LVDP levels compared to Exen 2 (*p* < 0.001). The regression analysis used to assess the effect of group and time on LVDP regarding the three main groups (Levo 3, Exen 2 and Combi) showed that the main effect of time was statistically significant (b = 1.452, *p* < 0.001); specifically, for every additional quarter, a 1.452 mmHg increase was observed. Levo 3 (*p* = 0.030) and Exen 2 (*p* = 0.015) presented lower LVDP levels compared to the Combi group.

### 3.4. Heart Rate (HR)

**Figure 6:**
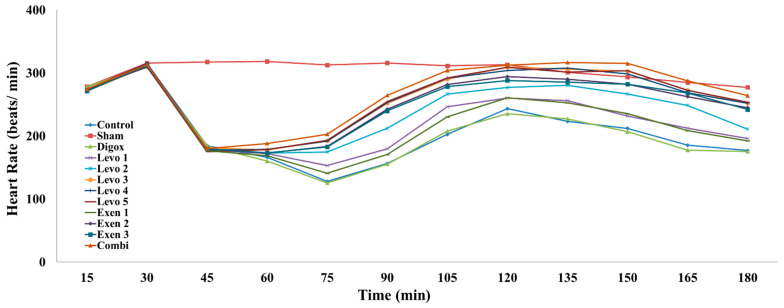
Changes in heart rate across groups over time.

Regarding the heart rate values, the main effect of time was found to be statistically significant (b = 0.878, *p* = 0.024). For every additional quarter, a 0.878 beats/min increase in the HR was observed. Sham, Levo 1, Levo 2, Levo 3, Levo 4, Levo 5, Exen 2, Exen 3 and Combi had higher HR levels compared to the control group (Table 4, Figure 6). Levo 1 presented lower HR compared to Levo 2 (*p* = 0.010), Levo 2 presented lower HR compared to Levo 3 (*p* = 0.002) and Exen 1 presented lower HR compared to Exen 2 (*p* < 0.001). Comparing the main groups, the main effect of time was found to be statistically significant (b = 4.458, *p* < 0.001); specifically, for every additional quarter, a 4.458 beats/min increase in HR was observed. Exen 2 presented lower HR compared to Combi (*p* = 0.016). Levo 3 also presented lower HR compared to Combi, but this was not statistically significant (*p* = 0.214).

### 3.5. Rate Pressure Product (RPP)

**Figure 7:**
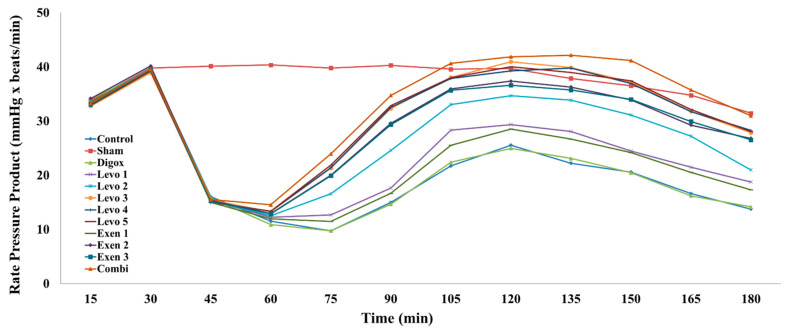
Changes in RPP across groups over time.

RPP is a parameter which demonstrates the stress put on the cardiac muscle based on the number of times it needs to beat per minute (HR) and the systolic blood pressure that it is pumping against (LVSP). The main effect of time here was found to be statistically insignificant (b = 0.103, *p* = 0.144). Sham, Levo 1, Levo 2, Levo 3, Levo 4, Levo 5, Exen 2, Exen 3 and Combi had significantly higher RPP compared to the control group (Table 5, Figure 7). Levo 1 presented lower RPP levels compared to Levo 2 (*p* = 0.003), Levo 2 lower compared to Levo 3 (*p* = 0.001) and Exen 1 lower compared to Exen 2 (*p* < 0.001). Regarding the main groups, the main effect of time was found to be statistically significant (b = 0.786, *p* < 0.001). It was observed that the groups Levo 3 (*p* = 0.050) and Exen 2 (*p* = 0.011) presented lower RPP levels compared to the Combi group.

### 3.6. Maximum Positive Value of the First Derivative of Pressure (+dP/dt)

**Figure 8:**
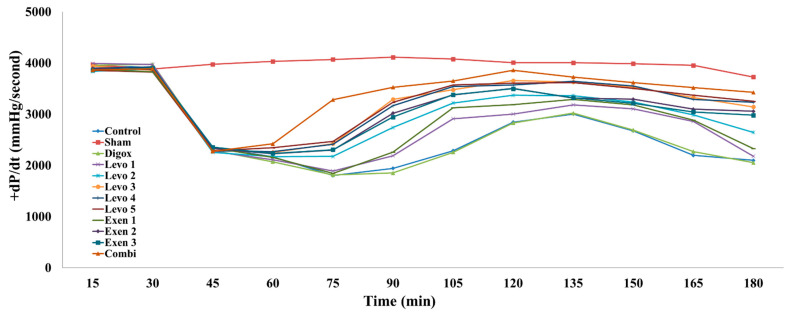
Changes in +dP/dt across groups over time.

+dP/dt is a sensitive indicator of changes in cardiac contractility given the fact that parameters such as preload, afterload and heart rate are well-controlled. The main effect of time between the +dP/dt and different types of groups was statistically significant (b = −15.652, *p* = 0.001). For every additional quarter, a 15.652 mmHg decrease in +dP/dt levels was observed. Sham, Levo 2, Levo 3, Levo 4, Levo 5, Exen 1, Exen 2, Exen 3 and Combi presented higher +dP/dt levels compared to the control group (Table 6, Figure 8). Levo 1 presented lower levels compared to Levo 2 (*p* = 0.033), Levo 2 presented lower levels compared to Levo 3 (*p* = 0.005) and Exen 1 presented lower +dP/dt compared to Exen 2 (*p* = 0.004). Additionally, the main effect of time between the main groups was found to be statistically significant (b = 17.944, *p* = 0.051). Levo 3 (*p* = 0.026) and Exen 2 (*p* = 0.018) presented lower +dP/dt levels compared to the Combi group.

### 3.7. Maximum Negative Value of the First Derivative of Pressure (−dP/dt)

**Figure 9:**
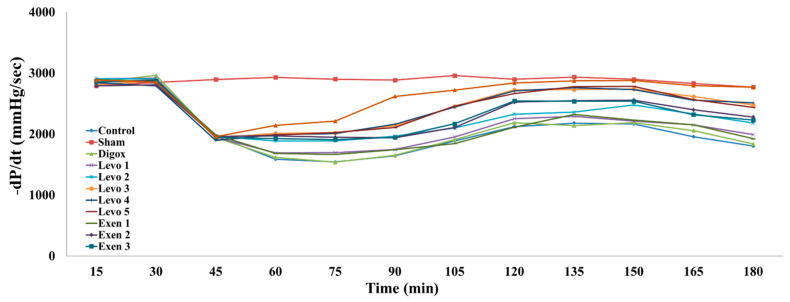
Changes in −dP/dt across groups over time.

Furthermore, the data showed that the main effect of time regarding −dP/dt was found to be statistically significant (b = −8.588, *p* = 0.003); specifically, for every additional quarter a 8.588 mmHg decrease in −dP/dt levels was observed. Sham, Levo 2, Levo 3, Levo 4, Levo 5, Exen 2, Exen 3 and Combi presented higher −dP/dt levels compared to the control group (Table 7, Figure 9). In addition, Levo 1 presented lower −dP/dt levels compared to Levo 2 (*p* = 0.011), Levo 2 presented lower −dP/dt levels compared to Levo 3 (*p* = 0.012) and the Exen 1 group presented lower levels compared to Exen 2 (*p* = 0.004). The main effect of time was found to be statistically significant for the main groups (b = 18.147, *p* < 0.001). It was observed that Levo 3 and Exen 2 presented lower −dP/dt levels compared to the combi group (*p* < 0.001).

### 3.8. Coronary Flow (CF)

**Figure 10:**
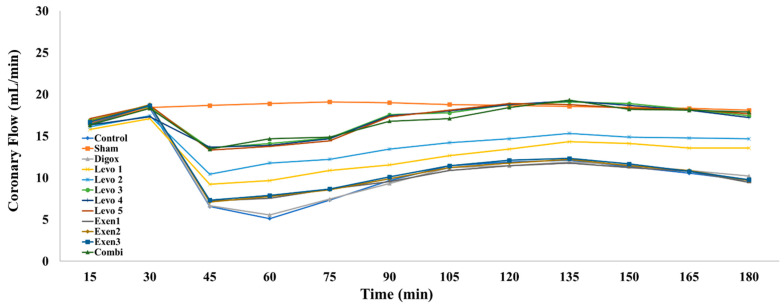
Changes in coronary flow across groups over time.

A further regression analysis was performed to determine whether there is association between the CF and different types of groups over time. The main effect of time was found to be non-statistically significant on CF levels (b = −0.004, *p* = 0.867). Sham, Levo 1, Levo 2, Levo 3, Levo 4, Levo 5 and Combi presented higher CF levels compared to the control group (Table 8, Figure 10). The Exen groups presented no significant difference to the control group. The pairwise comparison showed that Levo 1 presented lower CF levels compared to Levo 2 (*p* = 0.005) and Levo 2 lower levels compared to Levo 3 (*p* < 0.001). The analysis between the main groups showed that the main effect of time was statistically significant (b = 0.087, *p* = 0.032). Exen 2 presented lower CF levels compared to the Combi group (*p* < 0.001). There was no significant difference between the CF levels of Levo 3 and the Combi group (*p* = 0.787).

### 3.9. Arrhythmias

**Figure 11:**
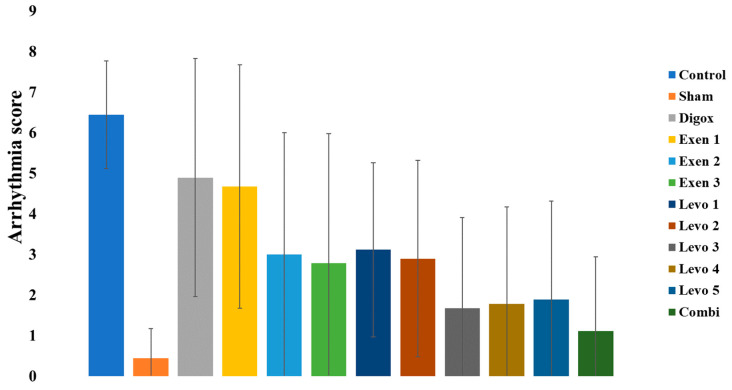
Differences in arrhythmias score across groups.

A one-way ANOVA was performed to assess possible differences in arrhythmia score among groups. There was a significant main effect of group (F = 4.798, *p* < 0.001) (Table 9 and Table 10). In order to examine whether there are any statistically significant differences between the main groups, extra pairwise comparison was conducted. The results showed that the control group presents higher score compared to Combi, Sham, Levo 1, Levo 2, Levo 3, Levo 4, Levo 5, Exen 2 and Exen 3. Moreover, no statistically significant differences were found between the Levo and Exen groups. Additionally, no statistically significant differences were found between the Combi group versus Levo 3 (*p* = 0.480) and Exen 2 (*p* = 0.091) (Figure 11).

### 3.10. Troponin I (cTnI)

**Figure 12:**
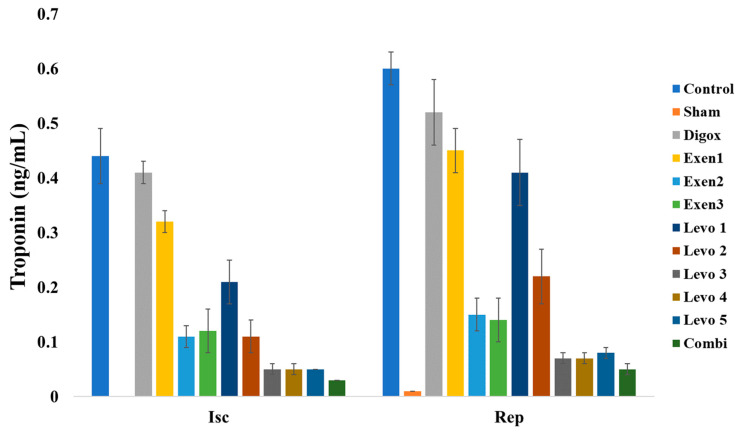
Changes in Troponin across groups over time.

Troponin is the most reliable and sensitive marker to assess the onset and evolution of the myocardial infarction. The main effect of time was found to be statistically significant on troponin levels (b = 0.073, *p* < 0.001). Sham, Digox, Levo 1, Levo 2, Levo 3, Levo 4, Levo 5, Exen 1, Exen 2, Exen 3 and Combi presented lower troponin levels compared to the control group (Table 11, Figure 12). Levo 1 presented higher levels compared to Levo 2 (*p* < 0.001), Levo 2 presented higher levels compared to Levo 3 (*p* < 0.001) and the Exen 1 presented higher levels compared to Exen 2 (*p* < 0.001). Regarding the main groups, the main effect of time was found to be statistically significant (b = 0.026, *p* < 0.001). Levo 3 (*p* = 0.010) and Exen 2 (*p* < 0.001) presented higher levels compared to Combi group.

### 3.11. Myocardial Creatine Kinase (CK-MB)

**Figure 13:**
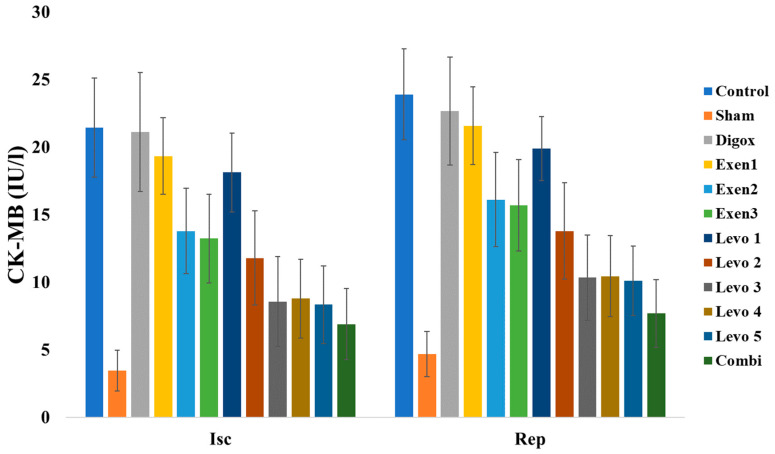
Changes in CK-MB across groups over time.

CK-MB is a classical marker of myocardial injury. The main effect of time was found to be statistically significant on CK-MB levels (b = 1.833, *p* < 0.001). Sham, Levo 1, Levo 2, Levo 3, Levo 4, Levo 5, Exen 1, Exen 2, Exen 3 and Combi presented lower CK-MB levels compared to the control group (Table 12, Figure 13). Levo 1 presented higher levels compared to Levo 2 (*p* < 0.001), Levo 2 presented higher levels compared to Levo 3 (*p* = 0.002) and Exen 1 presented higher levels than Exen 2 (*p* < 0.001). Finally, Exen 2 (*p* < 0.001) and Levo 3 presented higher levels than Combi (*p* = 0.011).

### 3.12. Lactate Dehydrogenase (LDH)

**Figure 14:**
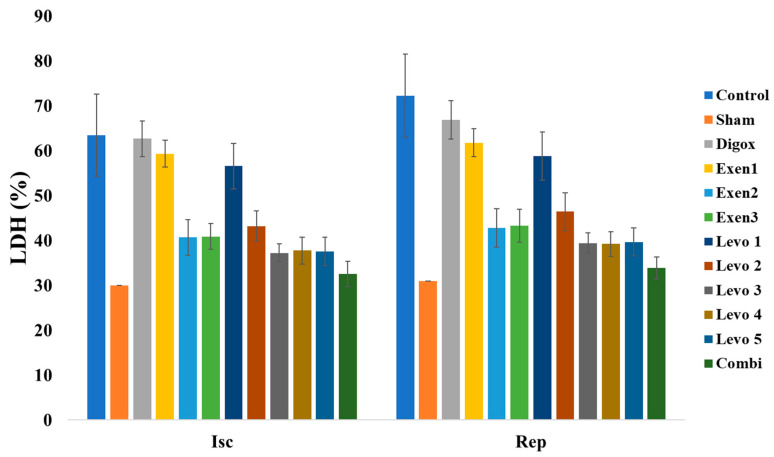
Changes in LDH across groups over time.

Regarding the LDH levels, the main effect of time was found to be statistically significant (b = 2.796, *p* < 0.001). Sham, Digox, Levo 1, Levo 2, Levo 3, Levo 4, Levo 5, Exen 1, Exen 2, Exen 3 and Combi presented lower LDH levels compared to the control group (Table 13, Figure 14). Additionally, Levo 1 presented higher LDH levels compared to Levo 2 (*p* < 0.001), Levo 2 presented higher levels than Levo 3 (*p* < 0.001) and Exen 1 higher levels than Exen 2 (0 < 0.001). Levo 3 and Exen 2 also present higher LDH levels compared to the combi group (*p* < 0.001).

### 3.13. Size of Myocardial Infarction

**Figure 15:**
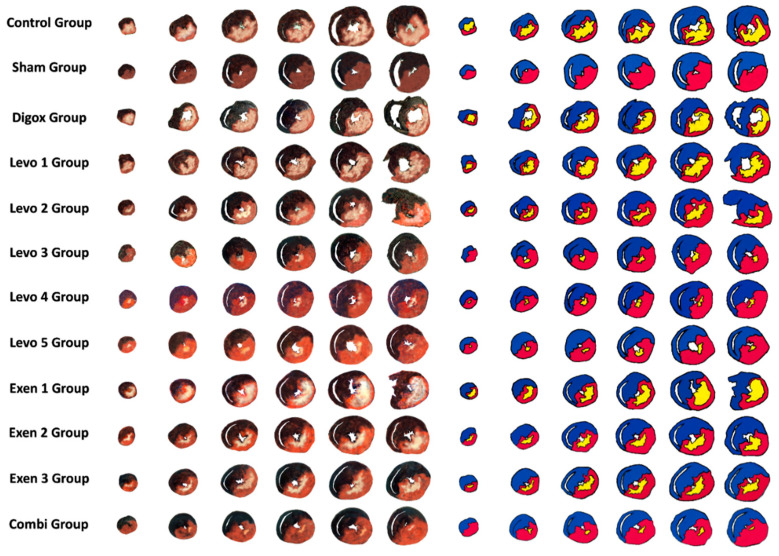
Sections of the hearts of all groups.

As mentioned before, the size of myocardial infarction was evaluated through the comparison of the infarct area to the area at risk. The risk area was also compared to the total area in order to secure the reproducibility of the infarction in all experiments. The representative sections of all groups in presented in Figure 15.

**Figure 16:**
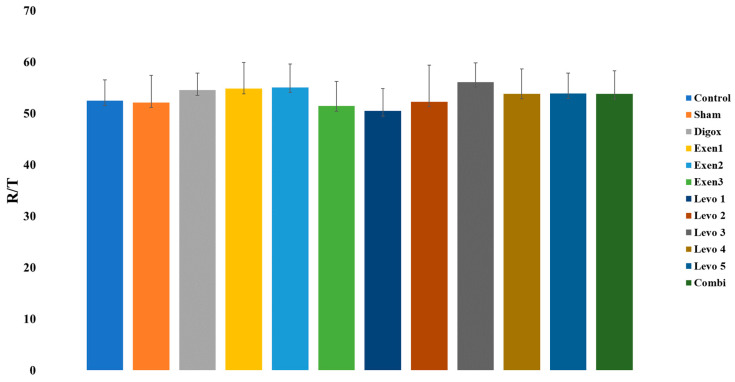
Changes in R/T across groups.

A one-way ANOVA was performed to assess possible differences of Risk Area/Total Area (R/T) among groups. There was not a significant main effect of group for Risk/Total (F = 1.109, *p* = 0.363). (Table 14 and Table 15, Figure 16).

**Figure 17:**
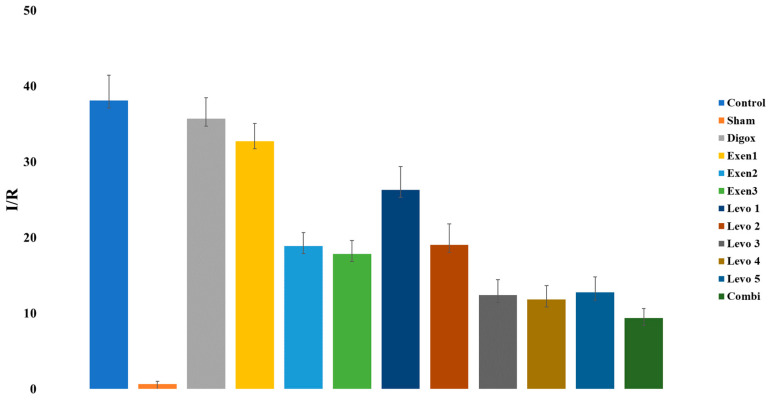
Changes in I/R across groups.

A one-way ANOVA was performed to assess possible differences of Ischemic Area/Risk Area (I/R) among groups. There was a significant main effect of group for I/R (F = 231.700, *p* < 0.001) (Table 16). In order to examine whether there are any statistically significant differences between the groups, extra pairwise comparison was conducted. The results showed that the control group presented higher I/R levels compared to Combi, Sham, Levo 1, Levo 2, Levo 3, Levo 4, Levo 5, Exen 1, Exen 2 and Exen 3 (Table 17). Additionally, results showed that Combi group presented lower I/R compared to Levo 3 (*p* = 0.005) and Exen 2 (*p* < 0.001). Moreover, Levo 1 presented higher I/R compared to Levo 2 (*p* < 0.001), Levo 2 higher I/R compared to Levo 3 (*p* < 0.001), Exen 1 higher I/R levels compared to Exen 2 (*p* < 0.001) (Figure 17).

### 3.14. Oxidative Stress

**Figure 18:**
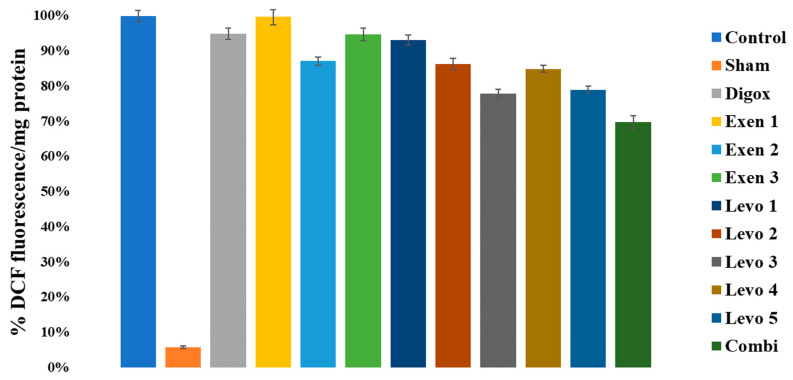
Differences in Reactive Oxygen Species across groups.

There was a significant main effect for Reactive Oxygen Species (ROS) (F = 37.091, *p* < 0.001) (Table 18). The results showed that the control group presented higher ROS levels compared to Combi, Sham, Levo 3 and Levo 5 (Table 19). Additionally, results showed that Combi presented lower ROS levels compared to Exen 2 (*p* = 0.005). There was no significant difference between Combi and Levo 3 (*p* < 0.999) The Exen 1 group presented higher ROS levels compared to Exen 2 (*p* = 0.037) There were no significant differences between the Levo groups (Figure 18).

## 4. Discussion

In this study, we tried to experimentally reproduce the evolution of regional ischemia followed by reperfusion in an isolated rat heart model. The negative control group (Digox) showed overall a non-significant improvement of the cardiac response to ischemia and reperfusion compared to the control group. Digoxin was used because it is a well-known agent used for the treatment of various cardiovascular diseases such as atrial fibrillation, atrial flutter and heart failure. The doses used were obtained to reflect average doses in similar studies in the literature [40,41,42,43,44,45,46], and were adjusted to our experimental protocol. Cardiac glycosides are used in the treatment of congestive heart failure. Its mechanism involves the inhibition of Na^+^/K^+^-ATPase and offers an increase in positive inotropy. The binding of glycosides to the catalytic α-subunit inhibits the sodium pump and increases intracellular Ca^2+^ availability for contractile proteins [40]. There are several experimental studies in the literature which show the positive inotropic effect of digoxin. Kang et al. showed that consecutive inhibition of first the α_2_- and then the α_1_-isoform of Na^+^/K^+^-ATPase mediates the positive inotropic effect of digoxin with increasing dosage in rat hearts [40]. D’Urso et al. demonstrated a cardioprotective effect of digoxin against ischemic injury in rat hearts and showed that it is independent of sodium pump inhibition, suggesting that it may be mediated by activation of a complex transduction pathway rather than by modulation of ionic homeostasis [41]. Singh et al. showed that the combined treatment with vitamin *p* and digoxin offered a significant effect against ischemia and reperfusion [42]. Duan et al. showed that a single bolus injection of ouabain or digoxin in the coronary tree at the beginning of reperfusion both improved significantly the recovery of left ventricular developed pressure and decreased LDH release, demonstrating a functional and structural protection [43]. In our study, the use of digoxin showed an improvement of haemodynamic parameters and the reduction of arrhythmias, but this improvement was not statistically significant compared to the control group. It did not offer any increase of the coronary flow. It also presented a significant decrease of TnI and LDH, but this effect was lower compared to both Levosimendan and Exenaditde groups. It did not offer any significant reduction neither of the size of myocardial infarction, nor of oxidative stress.

The groups treated with Levosimendan (Levo 1–5) showed a dose-dependent improvement of the haemodynamic parameters, the coronary flow, the occurrence of arrhythmias, the extension of myocardial injury, the size of the myocardial infarction and the amount of oxidative stress. After demonstrating the positive effect of Levosimendan, the next aim was to identify the doses with the maximal effect. This appeared to be in the group Levo 3 (0.1 μg/min). After increasing the doses in the groups Levo 4 and Levo 5, no significant further improvement was observed.

In the clinical practice, Levosimendan is already widely used for the treatment of acute and chronic heart failure. In cardiac surgery, it is often administrated preoperatively in patients with low ejection fractio, n significantly lowering mortality, with the benefit of having positive inotropic characteristics without impairing the diastolic function [47]. As mentioned before, several randomized clinical studies, retrospective analyses and metaanalyses have reported beneficial effects of Levosimendan regarding its inotropic, vasodilatatory and cardioprotective effect [10,11,12,13,14,15]. Many experimental studies have shown that a preconditioning induced by Levosimendan offers a cardioprotective effect. The exact mechanism of action is still not fully understood but several hypotheses have been suggested such as the activation of mitochondrial potassium (mK_ATP_+) channels [48,49,50] and phosphatidylinositol 3-kinase (PI3K) [51]. This cardioprotective action offers a decrease of infarct size and improves cardiac function after ischemia/reperfusion injury [48,52]. There are also many experimental studies in the current literature which have demonstrated the positive effects of Levosimendan in ischemia and reperfusion injury. Du Toit et.al showed that Levosimendan administrated either during ischemia alone or during ischemia and reperfusion improves reperfusion function and arrhythmias in the isolated guinea pig heart. The cardioprotective effects are considered independent of its vasodilatory properties and appear to be unrelated to its effects on ischemic tissue cAMP and cGMP levels. They supposed that the ATP-sensitive K_1_ channel-opening and possibly the phosphodiesterase (PDE) inhibitory properties of this compound may be implicated [52]. Kaheinen et al. showed that Levosimendan exerts a positive inotropic effect without disturbing the energy balance of the heart [53]. Another experimental model by Stehr et al. demonstrated that Levosimendan is an effective inotropic drug in ropivacaine-induced myocardial depression. They showed that disturbance of conductance and repolarization was not affected, but sinus bradycardia was reversed [54]. Ozturk et al. also demonstrated the cardioprotective effect of Levosimendan when administered before ischemia, protecting myocytes from ischemia-reperfusion induced apoptosis [55]. Zhou et al. showed that Celsior solution supplement with Levosimendan improved cardiac function recovery and reduced myocyte apoptosis in hypothermic preservation rat hearts. They supposed that the inhibition of cleavage of Bid through nitric oxide synthases (iNOS) and opening of mitochondrial KATP channel might be involved in the underlying mechanism [56]. Kiraz et al. demonstrated that that Levosimendan may be helpful in reducing myocardial necrosis, myocardial inflammation and myocardial tissue edema resulting from ischemia/reperfusion injury [57]. Moreover, Chen et al. showed that Levosimendan is superior to epinephrine in producing higher coronary flow levels and faster recovery when reversing bupivacaine-induced asystole [58]. Finally, Torregroza et al. demonstrated that cardioprotection under hyperglycemia can be restored by combining Levosimendan and Cyclosporine A (CsA) [59]. They also showed that infarct size could be reduced up to 56%. CsA alone had no effect on infarct size. The combination of Levosimendan and CsA induced a significant infarct size reduction compared to Levosimendan alone [59]. Stroethoff et al. presented a study in which Levosimendan reduced infarct size to 30 ± 5% [60]. This finding was also shown in the work of Bunte et al. [50]. They demonstrated that Levosimendan at a concentration of 0.3 μM reduced infarct size to 30 ± 7%. Higher concentrations with 1 μM Levosimendan did not confer stronger protection. This study showed that activation of mitochondrial large-conductance Ca^2+^-sensitive potassium (mBKCa) channels play a pivotal role in Levosimendan-induced preconditioning [50].

As far as Exenatide is concerned, the treated groups (Exen 1–3) also showed a dose-dependent improvement of the haemodynamic parameters and the occurrence of arrhythmias. Exenatide seemed not to have any significant effect increasing the coronary flow of the hearts. Exenatide offered some significant cardioprotection in terms of reducing myocardial injury as measured by the myocardial damage markers. It however did not show any significant reduction of the oxidative stress. Therefore, the possible cardioprotective mechanism of action probably does not include pathways involved in the oxidative stress. The doses presenting the maximal effect of the treatment here appeared to be in the group Exen 2 (0.01 μg/min). After increasing the doses in group Exen 3, no significant further improvement was observed. Overall, the effect of Exenatide seemed to be weaker compared to Levosimendan, especially concerning the prevention of arrhythmias and the reduction of myocardial infarction. Several experimental studies in the literature have implied a cardioprotective and inotropic effect of Exenatide apart from its well-known antidiabetic action. For instance, Ossum et al. were able to demonstrate a pharmacological postconditioning in the setting of an isolated heart, as illustrated by the cardioprotective and negative inotropic effects of GLP-1 [61]. Cao et al. managed to show that a pretreatment with exenatide significantly reduced cardiomyocyte apoptosis as measured by flow cytometry. In addition, exenatide inhibited excessive ROS production and maintained mitochondrial membrane potential. Furthermore, declined cytochrome-c release and cleaved caspase-3 expression and increased bcl-2 expression with concomitantly decreased Bax activation were observed in Exenatide-pretreated cultures. Their results suggest that Exenatide exerts a protective effect on cardiomyocytes, preventing tumor necrosis factor α (TNF-α)-induced apoptosis and its anti-apoptotic effects may be associated with protection of mitochondrial function [62]. Lee et al. expanded this result suggesting that Exenatide pre-treatment improves morphological and mechanical characteristics of mitochondria in response to ischemia-reperfusion injury in a rat model [63]. Zheng et al. offered further explanation by demonstrating that Exenatide improved cardiac function, increased translocation of glucose transporters (GLUTs) and suppressed translocation of fatty acid transporter FAT/CD36 after myocardial ischemia-reperfusion injury. This protective effect was mediated, at least in part, through modulation of the cardiac p38g mitogen-activated protein kinase MAPK [19].

The final endpoint of the study was the examination of a possible additive effect of the two agents. To our knowledge, there is to date no other study in the literature which examined the combination of those agents. After determining the groups where the maximal effect of those agents was observed (Levo 3 and Exen 2), both agents were coadministrated in those doses in the Combi group. The Combi group showed in all parameters a significant improvement compared to the control group and presented the highest performance among all groups. Finally, the Combi group was compared separately to Levo 3 and Exen 2 group, respectively. Compared to Levo 3 group, the Combi group showed a statistically significant improvement concerning the parameters LVDP (*p* = 0.003), RPP (*p* = 0.050), +dP/dt (*p* = 0.026) and −dP/dt (*p* < 0.001). No significant differences were observed in the parameters of the LVSP (*p* = 0.194), LVEDP (*p* = 0.128), CF (*p* = 0.787) and Arrhythmia scores (*p* = 0.619). Significant differences were also observed in the levels of TnI (*p* = 0.01), CK-MB (*p* = 0.036) and LDH (*p* < 0.001). As far as the size of myocardial infarction is concerned, there was also a significant superiority of the Combi group compared to Levo 3 (*p* = 0.005). There was, however, no significant difference between the two groups in terms of reducing the oxidative stress as both groups equally reduced the release of ROS (*p* < 0.999). Then, compared to Exen 2 group, it showed statistically improved values to the parameters of LVSP (*p* = 0.50), LVEDP (*p* = 0.019), LVDP (*p* = 0.015), HR (*p* = 0.016), RPP (*p* = 0.011), +dP/dt (*p* = 0.018) and CF (*p* < 0.001). No significant improvement was observed concerning the arrhythmia score (*p* = 0.093). As far as the coronary flow is concerned, this finding is obvious, as Exenatide did not contribute to the increase of the CF. Moreover, the Combi group performed a better decrease of myocardial damage as all markers were significantly lower; TnI: *p* < 0.001, CK-MB: *p* < 0.001, LDH *p* < 0.001. The reduction of infarct size was also significantly more effective in the Combi group (*p* < 0.001). The reduction of oxidative stress was obviously also significantly higher (*p* = 0.005), as Exenatide failed to present a significant reduction of oxidative stress.

Overall, the Combi group seems to have a superior haemodynamic improvement compared to both subgroups, as it provides a superior increase of CF compared to Exen 2, but not compared to Levo 3, and it offers no further improvement of arrhythmias compared to both groups. It presents a superior decrease of myocardial injury and the size of myocardial infarction. Finally, it offers a similar reduction of oxidative stress compared to Levo 3 and a significantly greater improvement compared to Exen 2 group. However, this superior effect cannot be pharmacologically defined as additive or synergistic. Whether these beneficial effects of simultaneous treatment were achieved by a lowered threshold of the same cellular mechanisms or parallel activation of different signaling pathways remains an open question. Investigating these exact underlying cellular mechanisms were beyond the scope of this study.

## 5. Conclusions

The administration of Levosimendan and Exenatide offers a significant benefit regarding the cardioprotection during ischemia and reperfusion. Moreover, the coadministration of both agents offers an even more beneficial outcome. The coadministration could offer a superior outcome in some clinical cases. It could also allow for the reduction of the doses of both agents in order to avoid the negative adverse effects in other cases. Further studies are certainly necessary in order to explain the underlying mechanism and to assess a possible clinical application of this coadministration.

## Figures and Tables

**Table 1:** Longitudinal analysis for LVSP for all groups.

	b	*p*	95% CI
Time	0.596	<0.001 *	0.402, 0.790
Group (Control: Ref. category)			
Sham	17.101	<0.001 *	13.814, 20.388
Digox	−0.414	0.805	−3.701, 2.873
Levo 1	2.630	0.117	−0.657, 5.917
Levo 2	9.550	<0.001 *	6.263, 12.837
Levo 3	14.150	<0.001 *	10.863, 17.437
Levo 4	14.113	<0.001 *	10.826, 17.400
Levo 5	14.012	<0.001 *	10.725, 17.299
Exen 1	0.991	0.555	−2.296, 4.278
Exen 2	12.935	<0.001 *	9.647, 16.222
Exen 3	12.599	<0.001 *	9.312, 15.886
Combi	16.527	<0.001 *	13.240, 19.815
Overall r-squared	0.234		
Chi-square	391.601	<0.001	
R-squared between	0.819		
R-squared within	0.029		

* *p* < 0.05.

**Table 2:** Longitudinal analysis for LVEDP for all groups.

	b	*p*	95% CI
Time	0.248	<0.001 *	0.128, 0.369
Group (Control: Ref. category)			
Sham	−11.776	<0.001 *	−13.814, −9.738
Digox	−1.174	0.259	−3.212, 0.864
Levo 1	−3.618	0.001 *	−5.656, −1.58
Levo 2	−4.100	<0.001 *	−6.138, −2.062
Levo 3	−5.681	<0.001 *	−7.719, −3.643
Levo 4	−5.836	<0.001 *	−7.874, −3.798
Levo 5	−6.087	<0.001 *	−8.125, −4.049
Exen 1	−3.149	0.002 *	−5.187, −1.111
Exen 2	−4.897	<0.001 *	−6.935, −2.859
Exen 3	−4.992	<0.001 *	−7.03, −2.954
Combi	−7.119	<0.001 *	−9.157, −5.081
Overall r-squared	0.134		
Chi-square	198.178	<0.001	
R-squared between	0.849		
R-squared within	0.013		

* *p* < 0.05.

**Table 3:** Longitudinal analysis for LVDP for all groups.

	b	*p*	95% CI
Time	0.348	0.011 *	0.081, 0.615
Group (Control: Ref. category)			
Sham	28.877	<0.001 *	24.361, 33.393
Digox	0.76	0.741	−3.755, 5.276
Levo 1	6.247	0.007 *	1.732, 10.763
Levo 2	13.65	<0.001 *	9.134, 18.165
Levo 3	19.831	<0.001 *	15.315, 24.347
Levo 4	19.949	<0.001 *	15.433, 24.464
Levo 5	20.098	<0.001 *	15.583, 24.614
Exen 1	4.14	0.072	−0.376, 8.656
Exen 2	17.831	<0.001 *	13.316, 22.347
Exen 3	17.591	<0.001 *	13.075, 22.107
Combi	23.646	<0.001 *	19.131, 28.162
Overall r-squared	0.226		
Chi-square	374.062	<0.001	
R-squared between	0.825		
R-squared within	0.005		

* *p* < 0.05.

**Table 4:** Longitudinal analysis for HR for all groups.

	b	*p*	95% CI
Time	0.878	0.024 *	0.114, 1.641
Group (Control: Ref. category)			
Sham	97.333	<0.001 *	83.737, 110.93
Digox	−2.324	0.738	−15.92, 11.272
Levo 1	16.667	0.016 *	3.07, 30.263
Levo 2	33.769	<0.001 *	20.172, 47.365
Levo 3	53.954	<0.001 *	40.357, 67.55
Levo 4	52.954	<0.001 *	39.357, 66.55
Levo 5	53.639	<0.001 *	40.043, 67.235
Exen 1	12.676	0.068	−0.92, 26.272
Exen 2	45.88	<0.001 *	32.283, 59.476
Exen 3	44.306	<0.001 *	30.709, 57.902
Combi	62.63	<0.001 *	49.033, 76.226
Overall r-squared	0.247		
Chi-square	383.947	<0.001	
R-squared between	0.798		
R-squared within	0.004		

* *p* < 0.05.

**Table 5:** Longitudinal analysis for RPP for all groups.

	b	*p*	95% CI
Time	0.103	0.144	−0.035, 0.242
Group (Control: Ref. category)			
Sham	17.311	<0.001 *	14.876, 19.746
Digox	−0.038	0.976	−2.473, 2.397
Levo 1	2.869	0.021 *	0.434, 5.304
Levo 2	6.403	<0.001 *	3.968, 8.839
Levo 3	10.283	<0.001 *	7.848, 12.718
Levo 4	10.186	<0.001 *	7.750, 12.621
Levo 5	10.293	<0.001 *	7.858, 12.729
Exen 1	2.052	0.099	−0.384, 4.487
Exen 2	8.788	<0.001 *	6.353, 11.224
Exen 3	8.501	<0.001 *	6.065, 10.936
Combi	12.325	<0.001 *	9.890, 14.761
Overall r-squared	0.253		
Chi-square	404.098	<0.001	
R-squared between	0.807		
R-squared within	0.002		

* *p* < 0.05.

**Table 6:** Longitudinal analysis for +dP/dt for all groups.

	b	*p*	95% CI
Time	−15.652	0.001 *	−25.188, −6.115
Group (Control: Ref. category)			
Sham	1380.55	<0.001 *	1128.166, 1632.927
Digox	−12.000	0.926	−264.381, 240.381
Levo 1	203.120	0.115	−49.260, 455.501
Levo 2	388.194	0.003 *	135.814, 640.575
Levo 3	632.796	<0.001 *	380.416, 885.177
Levo 4	629.120	<0.001 *	376.740, 881.501
Levo 5	641.537	<0.001 *	389.156, 893.918
Exen 1	256.556	0.046 *	4.175, 508.936
Exen 2	504.565	<0.001 *	252.184, 756.946
Exen 3	484.481	<0.001 *	232.101, 736.862
Combi	819.093	<0.001 *	566.712, 1071.473
Overall r-squared	0.254		
Chi-square	203.746	<0.001	
R-squared between	0.668		
R-squared within	0.009		

* *p* < 0.05.

**Table 7:** Longitudinal analysis for −dP/dt for all groups.

	b	*p*	95% CI
Time	−8.588	0.003 *	−14.330, −2.845
Group (Control: Ref. category)			
Sham	829.824	<0.001 *	718.475, 941.173
Digox	24.519	0.666	−86.831, 135.868
Levo 1	97.454	0.086	−13.895, 208.803
Levo 2	224.704	<0.001 *	113.355, 336.053
Levo 3	412.426	<0.001 *	301.077, 523.775
Levo 4	400.870	<0.001 *	289.521, 512.220
Levo 5	409.741	<0.001 *	298.392, 521.090
Exen 1	68.639	0.227	−42.710, 179.988
Exen 2	269.657	<0.001 *	158.308, 381.007
Exen 3	267.120	<0.001 *	155.771, 378.470
Combi	579.620	<0.001 *	468.271, 690.970
Overall r-squared	0.294		
Chi-square	418.714	<0.001	
R-squared between	0.81		
R-squared within	0.007		

* *p* < 0.05.

**Table 8:** Longitudinal analysis for coronary flow for all groups.

	b	*p*	95% CI
Time	−0.004	0.867	−0.053, 0.044
Group (Control: Ref. category)			
Sham	7.583	<0.001	6.761, 8.405
Digox	0.083	0.842	−0.739, 0.905
Levo 1	2.111	<0.001	1.289, 2.933
Levo 2	3.287	<0.001	2.465, 4.109
Levo 3	6.259	<0.001	5.437, 7.081
Levo 4	6.065	<0.001	5.243, 6.887
Levo 5	6.176	<0.001	5.354, 6.998
Exen 1	0.259	0.536	−0.563, 1.081
Exen 2	0.463	0.269	−0.359, 1.285
Exen 3	0.574	0.171	−0.248, 1.396
Combi	6.083	<0.001	5.261, 6.905
Overall r-squared	0.466		
Chi-square	323.674	<0.001	
R-squared between	0.000		
R-squared within	0.771		

**Table 9:** Effect of group on arrhythmias score.

Arrhythmias Score	Mean	SD	F	*p*-Value
Combi	1.11	1.83	4.798	<0.001
Sham	0.44	0.73		
Digox	4.89	2.93		
Levo 1	3.11	2.15		
Levo 2	2.89	2.42		
Levo 3	1.67	2.24		
Levo 4	1.78	2.39		
Levo 5	1.89	2.42		
Exen 1	4.67	3.00		
Exen 2	3.00	2.55		
Exen 3	2.78	3.19		
Control	6.44	1.33		

**Table 10:** Pairwise comparisons for arrhythmias score among groups.

	Arrhythmias Score	Mean Difference	*p*-Value
Control	Combi	5.33	<0.001
	Sham	6.00	<0.001
	Digox	1.56	0.166
	Levo 1	3.33	0.004
	Levo 2	3.56	0.002
	Levo 3	4.78	<0.001
	Levo 4	4.67	<0.001
	Levo 5	4.56	<0.001
	Exen 1	1.78	0.114
	Exen 2	3.44	0.003
	Exen 3	3.67	0.001

**Table 11:** Longitudinal analysis for Troponin for all groups.

	b	*p*	95% CI
Time	0.073	<0.001	0.061, 0.086
Group (Control: Ref. category)			
Sham	−0.514	<0.001	−0.545, −0.484
Digox	−0.054	0.001	−0.084, −0.024
Levo 1	−0.207	<0.001	−0.237, −0.177
Levo 2	−0.355	<0.001	−0.386, −0.325
Levo 3	−0.460	<0.001	−0.49, −0.429
Levo 4	−0.463	<0.001	−0.493, −0.433
Levo 5	−0.457	<0.001	−0.487, −0.427
Exen 1	−0.136	<0.001	−0.166, −0.106
Exen 2	−0.390	<0.001	−0.420, −0.360
Exen 3	−0.393	<0.001	−0.423, −0.363
Combi	−0.480	<0.001	−0.510, −0.450
Overall r-squared	0.94		
Chi-square	3155.101	<0.001	
R-squared within	0.532		
R-squared between	0.974		

**Table 12:** Longitudinal analysis for CK-MB for all groups.

	b	*p*	95% CI
Time	1.833	<0.001	1.020, 2.646
Group (Control: Ref. category)			
Sham	−18.611	<0.001	−20.602, −16.620
Digox	−0.778	0.442	−2.769, 1.214
Levo 1	−3.667	<0.001	−5.658, −1.675
Levo 2	−9.889	<0.001	−11.880, −7.898
Levo 3	−13.222	<0.001	−15.214, −11.231
Levo 4	−13.056	<0.001	−15.047, −11.064
Levo 5	−13.444	<0.001	−15.436, −11.453
Exen 1	−2.222	0.029	−4.214, −0.231
Exen 2	−7.722	<0.001	−9.714, −5.731
Exen 3	−8.222	<0.001	−10.214, −6.231
Combi	−15.389	<0.001	−17.380, −13.398
Overall r-squared	0.803		
Chi-square	801.788	<0.001	
R-squared within	0.793		
R-squared between	0.804		

**Table 13:** Longitudinal analysis for LDH for all groups.

	b	*p*	95% CI
Time	2.796	<0.001	1.668, 3.924
Group (Control: Ref. category)			
Sham	−37.333	<0.001	−40.097, −34.57
Digox	−3.056	0.030	−5.819, −0.292
Levo 1	−10.167	<0.001	−12.930, −7.403
Levo 2	−23.000	<0.001	−25.763, −20.237
Levo 3	−29.500	<0.001	−32.263, −26.737
Levo 4	−29.333	<0.001	−32.097, −26.57
Levo 5	−29.222	<0.001	−31.985, −26.459
Exen 1	−7.278	<0.001	−10.041, −4.515
Exen 2	−26.111	<0.001	−28.874, −23.348
Exen 3	−25.722	<0.001	−28.485, −22.959
Combi	−34.611	<0.001	−37.374, −31.848
Overall r-squared	0.900		
Chi-square	1083.66	<0.001	
R-squared between	0.907		
R-squared within	0.571		

**Table 14:** Effect of group on R/T.

R/T	Mean	SD	F	*p*-Value
Combi	53.76	4.51	1.109	0.363
Sham	52.11	5.29		
Digox	54.50	3.31		
Levo 1	50.46	4.36		
Levo 2	52.26	7.15		
Levo 3	56.10	3.70		
Levo 4	53.82	4.86		
Levo 5	53.90	3.96		
Exen 1	54.81	5.07		
Exen 2	55.07	4.54		
Exen 3	51.41	4.78		
Control	52.48	4.01		

**Table 15:** Pairwise comparisons for R/T among groups.

	R/T	Mean Difference	*p*-Value
Control	Combi	−1.29	<0.999
	Sham	0.37	<0.999
	Digox	−2.02	<0.999
	Levo 1	2.01	<0.999
	Levo 2	0.22	<0.999
	Levo 3	−3.63	<0.999
	Levo 4	−1.35	<0.999
	Levo 5	−1.43	<0.999
	Exen 1	−2.33	<0.999
	Exen 2	−2.59	<0.999
	Exen 3	1.07	<0.999

**Table 16:** Effect of group on I/R.

I/R	Mean	SD	F	*p*-Value
Combi	9.37	1.25	231.700	<0.001
Sham	0.66	0.35		
Digox	35.71	2.75		
Levo 1	26.28	3.10		
Levo 2	19.04	2.78		
Levo 3	12.41	2.03		
Levo 4	11.84	1.79		
Levo 5	12.74	2.06		
Exen 1	32.73	2.34		
Exen 2	18.89	1.75		
Exen 3	17.83	1.79		
Control	38.09	3.37		

**Table 17:** Pairwise comparisons for Ischemia/R among groups.

	I/R	Mean Difference	*p*-Value
Control	Combi	28.72	<0.001
	Sham	37.42	<0.001
	Digox	2.38	<0.999
	Levo 1	11.80	<0.001
	Levo 2	19.05	<0.001
	Levo 3	25.67	<0.001
	Levo 4	26.25	<0.001
	Levo 5	25.35	<0.001
	Exen 1	5.35	<0.001
	Exen 2	19.20	<0.001
	Exen 3	20.26	<0.001

**Table 18:** Effect of group on Reactive Oxygen Species.

ROS	Mean	SD	F	*p*-Value
Combi	10.19	1.81	37.091	<0.001
Sham	0.84	0.40		
Digox	13.87	1.67		
Levo 1	13.60	1.39		
Levo 2	12.61	1.62		
Levo 3	11.38	1.24		
Levo 4	12.41	1.04		
Levo 5	11.54	0.99		
Exen 1	14.57	2.26		
Exen 2	12.73	1.16		
Exen 3	13.84	1.74		
Control	14.61	1.72		

**Table 19:** Pairwise comparisons for Reactive Oxygen Species among groups.

	ROS	Mean Difference	*p*-Value
Control	Combi	4.42	<0.001
	Sham	13.77	<0.001
	Digox	0.75	<0.999
	Levo 1	1.01	<0.999
	Levo 2	2.01	<0.999
	Levo 3	3.24	0.026
	Levo 4	2.21	0.013
	Levo 5	3.07	0.048
	Exen 1	0.05	<0.999
	Exen 2	1.89	<0.999
	Exen 3	0.78	<0.999

## Data Availability

The data presented in this study are available on request from the corresponding author.
